# Early Localization of Bronchogenic Carcinoma

**DOI:** 10.1155/DTE.1.75

**Published:** 1994

**Authors:** S. Lam, C. Macaulay, J. C. Leriche, N. Ikeda, B. Palcic

**Affiliations:** 1 Cancer Imaging British Columbia Cancer Research Centre and The University of British Columbia Vancouver B.C. V5Z 1L3 Canada; 2 Laboratory Medicine British Columbia Cancer Agency Vancouver B.C. V5Z 4E6 Canada; 3 Department of Surgery Tokyo Medical College Hospital Tokyo Japan; 4 Cancer Imaging British Columbia Cancer Research Centre 601 West 10th Avenue Vancouver B.C. V5Z 1L3 Canada

## Abstract

The performance of a fluorescence imaging device was compared with conventional white-light bronchoscopy in 100 patients with lung cancer, 46 patients with resected stage I non-small cell lung cancer, 10 patients with head and neck cancer, and 67 volunteers who had smoked at least 1 pack of cigarettes per day for 25 years or more. Using differences in tissue autofluorescence between premalignant, malignant, and normal tissues, fluorescence bronchoscopy was found to detect significantly more areas with moderate/severe dysplasia or carcinoma *in situ* than conventional white-light bronchoscopy with a similar specificity. Multiple foci of dysplasia or cancer were found in 13–24% of these individuals. Fluorescence bronchoscopy may be an important adjunct to conventional bronchoscopic examination to improve our ability to detect and localize premalignant and early lung cancer lesions.


*Diagnostic and Therapeutic Endoscopy*, Vol. 1, pp. 75–78
Reprints available directly from the publisher
Photocopying permitted by license only

© 1994 Harwood Academic Publishers GmbH
Printed in Malaysia

### Early Localization of Bronchogenic Carcinoma

S. LAM[1], C. MACAULAY[1], J. C. LERICHE[2], N. IKEDA[3], and B. PALCIC[1]

*Cancer Imaging, British Columbia Cancer Research Centre and The University of British Columbia, Vancouver, B.C. V5Z 1L3 Canada;[1] Laboratory Medicine, British Columbia Cancer Agency, Vancouver, B.C. V5Z 4E6, Canada;[2] Department of Surgery, Tokyo Medical College Hospital, Tokyo, Japan[3]*

*(April 4, 1994; in final form May 20, 1994)*

The performance of a fluorescence imaging device was compared with conventional white-light bronchoscopy in 100 patients with lung cancer, 46 patients with resected stage I non-small cell lung cancer, 10 patients with head and neck cancer, and 67 volunteers who had smoked at least 1 pack of cigarettes per day for 25 years or more. Using differences in tissue autofluorescence between premalignant, malignant, and normal tissues, fluorescence bronchoscopy was found to detect significantly more areas with moderate/severe dysplasia or carcinoma *in situ* than conventional white-light bronchoscopy with a similar specificity. Multiple foci of dysplasia or cancer were found in 13–24% of these individuals. Fluorescence bronchoscopy may be an important adjunct to conventional bronchoscopic examination to improve our ability to detect and localize premalignant and early lung cancer lesions.

**KEY WORDS:** autofluorescence, bronchoscopy, dysplasia, early lung cancer

### INTRODUCTION

The best results of photodynamic therapy (PDT) for lung cancer are seen in patients with carcinoma *in situ* or microinvasive cancers. Complete eradication of these early lung cancer lesions without loss of normal lung tissue or lung function capacity can be seen in over 90% of these patients (Hayata *et al.*, 1993; Furuse *et al.*, 1993; Edell and Cortese, 1992). When precancerous lesions are found, chemoprevention agents, such as 13-*cis*-retinoic acid or Retinol can be used to regress the lesions (Lippman *et al.*, 1990, Pastorino *et al.*, 1993). Despite the availability of these treatment modalities, very few patients benefit from them because dysplasia and early lung cancer lesions are very difficult to detect and localize with conventional white-light bronchoscopic examination. In a study by Woolner and coworkers (Woolner *et al.*, 1984), only 29% of carcinoma *in situ* (CIS) were visible to an experienced endoscopist. Even for pathologists who had the opportunity to carefully examine the resected specimens, they were able to visualize the site of the CIS lesions in only 41% of the cases (Woolner *et al.*, 1984). In an attempt to overcome this problem, a lung imaging fluorescence endoscopic (LIFE) device was developed to detect precancerous and CIS lesions using differences in tissue autofluorescence between normal and abnormal tissues (Hung *et al.*, 1991; Lam *et al.*, 1993; Palcic *et al.*, 1991; Lam and Palcic, 1993).

The objective of this study was to determine if fluorescence bronchoscopy using the LIFE device can improve the ability of conventional white-light bronchoscopy to detect bronchial dysplasia and CIS.

### MATERIALS AND METHODS

### Life

LIFE is comprised of a helium-cadmium laser as a light source (442 nm), two image-intensified CCD cameras with green (520 nm) and red (>630 nm) filters, respectively, a computer with an imaging board, and a color video monitor (Lam and Palcic, 1993). Two images at different (red and green) wavelengths are simultaneously captured in precise registration by the imaging board. The images

Address for correspondence: Dr. Stephen Lam, Cancer Imaging, British Columbia Cancer Research Centre, 601 West 10th Avenue, Vancouver B.C., V5Z 1L3, Canada.

are then combined and processed by an imaging board using a specially developed algorithm that allows normal tissue to be clearly distinguished from malignant tissue when displayed as a pseudocolor image on the video monitor. The computed image is displayed in real time (at video rates). The detection of lung tumors is based on the observation that tumors *in vivo* have considerably lower autofluorescence in the green than normal tissue, while emission in the red is similar (not as reduced) (Hung *et al.*, 1991). The computed image is independent of the distance between the bronchoscope tip and the bronchial wall. The processed image can be displayed as desired, for example, normal tissue as green and tumor as brown or brownish red (Fig. 1A and B). An abnormal area can be biopsied under direct vision for pathologic confirmation.

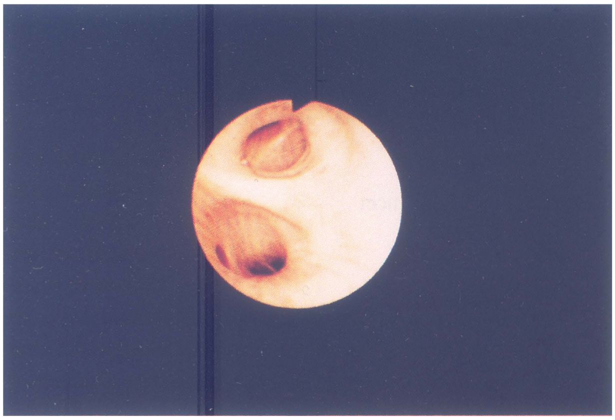

**Figure 1A** Left upper lobe under white-light bronchoscopy, no abnormality as found.

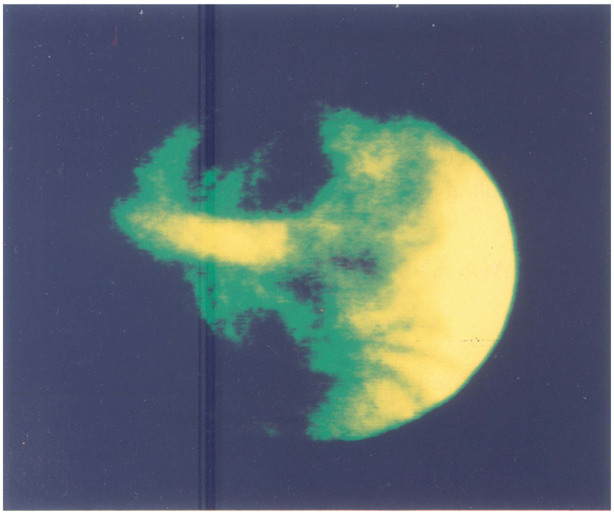

**Figure 1B** Same area as 1A under fluorescence imaging with the LIFE device. A small area of carcinoma *in situ* was found. Autofluorescence from normal bronchial tissue was represented by green color. Carcinoma *in situ* appeared reddish-brown.

### SUBJECTS

Four groups of subjects were studied. Group I consisted of 100 patients with lung cancer (age 63 ± 9 years, male/female, 67/33). The pathology of the primary lung cancer was squamous 50%, adenocarcinoma 25%, large cell carcinoma 16%, small cell carcinoma 7%, and 2% other tumor types. Group II consisted of 46 patients with stage I completely resected lung cancer (age 66 ± 9 years, male/female, 34/12). Group III consisted of 10 patients with head and neck cancer (age 62 ± 10 years, all males). Group IV consisted of 67 volunteers who had smoked more than 1 pack of cigarettes per day for 25 years or more (age 56 ± 8 years), male/female, 51/16). There were 48 current smokers and 19 ex-smokers. They had smoked an average of 50 ± 27 pack years.

### CLINICAL PROTOCOL

Conventional fiberoptic bronchoscopy was performed with patients under local anesthesia. White-light examination was first done with an Olympus BF20D fiberoptic bronchoscope. Areas suspected of dysplasia or cancer were recorded and noted as such for subsequent biopsy. Fluorescence bronchoscopy with the LIFE device was then carried out biopsy specimens for pathologic examination were taken of all abnormal areas discovered by the white-light or the fluorescence examination, or both. In addition, two or more random biopsies were done in the visually (white-light and fluorescence) normal areas. On the average, the fluorescence examination and extra biopsies added another 5 to 10 minutes to a standard fiberoptic bronchoscopic procedure.

This study was approved by the Clinical Investigations Review Committee of the University of British Columbia and the British Columbia Cancer Agency.

**Table 1** Prevalence of Dysplasia and Carcinomas *in situ*

| Group | *No. of subjects* | *Dysplasia* Moderate | *Dysplasia* Severe | *Carcinoma In Situ* | *≥ 2 Foci* |
|---|---|---|---|---|---|
| I | 100 | 14% | 11% | 15% | 15% |
| II | 46 | 18% | 4% | 13% | 24% |
| III | 10 | 20% | 10% | 10% | 20% |
| IV | 67 | 36% | 15% | 5% | 13% |

Group I = Known/suspected lung cancer; group II = stage I completely resected lung cancer; group III = head and neck cancer; group IV = volunteer smokers.

**Table 2** Bronchoscopy and Biopsy Results

| | Normal/Inflamed: *Fluorescence* − | Normal/Inflamed: *Fluorescence* + | Moderate/severe Dysplasia: *Fluorescence* − | Moderate/severe Dysplasia: *Fluorescence* + | Carcinoma *in situ*: *Fluorescence* − | Carcinoma *in situ*: *Fluorescence* + |
|---|---|---|---|---|---|---|
| White Light − | 275 | 33 | 15 | 33 | 3 | 18 |
| White Light + | 18 | 12 | 6 | 24 | 0 | 14 |

### DATA ANALYSES

The principle objective of this study was to measure the increase in detection rate for moderate dysplasia or worse achieved by the addition of LIFE to white-light bronchoscopy using the bronchial biopsy pathology as the "gold" standard. The positive rates among the two modes of examination were compared using the $X^2$ test. The specificity of white light versus fluorescence bronchoscopy was also compared.

### RESULTS

From the 223 subjects, 338 biopsies were found to have a normal/inflamed histopathology, 78 biopsies were found to show moderate or severe dysplasia and 35 were found to have carcinoma *in situ*.

The prevalence of dysplasia and carcinoma *in situ* in the four study groups are shown in Table 1.

Of the CIS lesions that were detected by white-light examination, 84% were superficial and 16% were nodular/polyploid.

The proportion of biopsy proven moderate/severe dysplasia, and CIS detected by white-light bronchoscopy alone was 38.5% and 40%, respectively. With the addition of LIFE examination, the detection rate was 73.1 and 91.4% respectively. The specificity of white-light bronchoscopy was 91.1%. With fluorescence bronchoscopy, the specificity was 86.7% (Table 2).

### DISCUSSION

Dysplasia and carcinoma *in situ* are difficult to detect by conventional bronchoscopy because these lesions are only a few cell layers thick (0.2 to 1 mm) and a few millimeters in surface diameter (Hayata *et al.*, 1993; Woolner *et al.*, 1984; Woolner 1983; Nagamoto *et al.*, 1986). Because of this, they may not produce any visible abnormality on conventional white-light bronchoscopy. The superficial or flat type of early lung cancer usually presents as areas of paleness, roughness, redness, microgranularity, or loss of luster in the surface mucosa. The mucosal folds or bronchial bifurcation may appear to be swollen or thickened with the loss of clarity. They are not usually detectable by bronchoscopy until they are 0.5 cm or more in diameter (Hayata *et al.*, 1993; Nagamoto *et al.*, 1986). The nodular or polypoid type of early lung cancer usually presents as an area of protrusion. Nodular lesions are easier to detect because they are elevated from the adjacent normal mucosa. Lesions as small as 0.2 cm can be detected by white-light bronchoscopy (Hayata *et al.*, 1993; Nagamoto *et al.*, 1986). The bronchoscopic appearance of bronchial dysplasia has not been carefully studied. In this study, the dysplastic lesions that were visible on white-light bronchoscopy presented as focal thickening of the mucosal fold or bifurcation. Overall, 40% of the dysplastic lesions or CIS were visible by white-light examination. Since the bronchoscope only serves as a means to examine the surface of a body cavity, it is unlikely that further advances in white-light bronchoscopy can improve our ability to detect pre-cancerous lesions or early cancer. Our study suggests that instead of making use of the reflected/scattered light, one can use light induced tissue autofluorescence to improve the detection rate of these early lung cancer lesions by more than two times with a similar specificity.

The results in this study are similar to our previous study involving 94 subjects where we observed a sensitivity of 72.5% for fluorescence bronchoscopy in detecting moderate/severe dysplasia and carcinoma *in situ* with a specificity of 94%. Using white light bronchoscopy, the sensitivity was 48.4% with the same specificity (Lam *et al.*, 1993). In both studies, false-positives were due to inflammation in the submucosa or squamous metaplasia resulting in swelling or thickening of the mucosa. The reason for the false-negative sites (3/35 CIS lesions) was not clear. All three tumors regressed spontaneously on follow-up. It is possible that *in situ* carcinomas with nonaggressive biological behavior have different reflectance and fluorescence properties.

The concept of fluorescence detection has intrigued the minds of many since the beginning of the twentieth century. In 1924, Policard observed that in an experimental model of sarcoma, the tumor tissue fluoresced red upon illumination by Wood's light (Policard, 1924). In 1933, Sutro and Burman observed that when surgically excised breast tissue was exposed to Wood's light, normal breast tissue fluoresced green, while breast cancer tissues fluoresced purple (Sutro ar. Burman, 1993). These observations were confirmed by others in cancer of the skin and mouth in addition to breast cancer (Ronchese, 1954). Red fluorescence was found to be associated with advanced cancers only (Ronchese, 1954). The color of the natural tissue autofluorescence induced by filtered ultraviolet light is variable and in addition, the emitted light is of very low intensity. For these reasons, it has been very difficult to observe it visually, and therefore, almost all of the research in fluorescence bronchoscopy since the 1960s employed exogenous fluorescent compounds to enhance humans ability to detect early lung cancer. It was not until the advent of image-intensified cameras and computer image processing technologies that tissue autofluorescence alone could be used for detecting small thin early cancers and premalignant lesions.

Synchronous or second primary cancers occur commonly in patients with lung cancer. An autopsy study of Auerbach and co-workers showed that in patients who died of lung cancer, CIS could be found in 15% of these patients (Auerbach *et al.*, 1961). In the same study, in smokers who died of non-lung cancer causes, CIS was found in 4.3% of those who smoked 1–2 packs per day and 11.4% of those who smoked more than 2 packs a day (Auerbach *et al.*, 1961). In patients resected stage I lung cancer, second primary tumors occur in 10–20% of cases (Pastorino *et al.*, 1993; Thomas *et al.*, 1990; Cortese, *et al.*, 1983). The prevalence of dysplasia and CIS observed in our patients with lung cancer and in the smoking volunteers are consistent with the findings in these earlier studies.

Our study suggests that fluorescence bronchoscopy, in conjunction with standard white-light bronchoscopy, may be very useful in the detection of synchronous and second primary tumors in patients with lung cancer. In high risk populations such as heavy smokers, fluorescence bronchoscopy may also be useful for localizing precancerous lesions and CIS. This technology, in combination with local treatments such as photodynamic therapy which can eradicate these lesions without loss of normal lung tissues, offers hope that the traditionally poor prognosis of lung cancer may be altered in a significant way.

### ACKNOWLEDGMENTS

This study was supported by the National Cancer Institute of Canada, the Pacific Pulmonary Research Society, and Xillix Technologies Corp., Vancouver, Canada

### REFERENCES

Auerbach, O., Stout, A. P., Hammond, E. C. and Garfinkel, L. (1961) Changes in bronchial epithelium in relation to cigarette smoking and in relation to lung cancer. *N. Engl. J. Med.*, 265:253–267.

Cortese, D. A., Pairolero, P. D., Bergstralh, E. J. B. *et al.* (1983) Roentgenographically occult lung cancer: A ten year experience. *J. Thorac. Cardiovasc. Surg.*, 86:373–80.

Edell, E. S., Cortese, D. A. (1992) Photodynamic therapy in the management of early superficial squamous cell carcinoma as an alternative to surgical resection. *Chest* 102:1319–1322.

Furuse, K., Fukuoka, M., Kato H. *et al.* (1993) A prospective study on photodynamic therapy with Photofrin II for centrally located early-stage lung cancer. *J. Clin. Oncol.*, 11:1852–1857.

Hayata Y., Kalo, H., Konaka, C., Okunaka, T. (1993) Photodynamic therapy in early stage lung cancer. *Lung Cancer*, 9:287–294.

Hung, J., Lam, S., LeRiche, J. C., Palcic B. (1991) Autofluorescence of normal and malignant bronchial tissue. *Lasers Surg. Med.*, 11:99–105.

Lam, S., MacAulay C., Hung, J. *et al.* (1993) Detection of dysplasia and carcinoma *in-situ* using a lung imaging fluorescence endoscope (LIFE) device. *J. Thorac. Cardiovasc. Surg.*, 105:1035–1040.

Lam, S. and Palcic, B. (1993) Fluorescence detection. In: Roth, J. A., Cox, J. D., Hong, W. K. (eds.) *Lung Cancer*, Blackwell Scientific Publications 325–338.

Lippman, S. M., Lee, J. S. and Lotan, R. *et al.* (1990) Chemoprevention of upper aerodigestive tract cancers: A report of the Third Upper Aerodigestive Cancer Task Force Workshop. Head and Neck 12:5–20.

Nagamoto, N., Saito, Y., Iami, T. *et al.* (1986) Roentgenographically occult squamous cell carcinoma: Location in the bronchi, depth of invasion and length of axial involvement of the bronchus. *Tohoku J. Exp. Med.*, 148:241–256.

Palcic B., Lam, S., Hung J. and MacAulay C. (1991) Detection and localization of early lung cancer by imaging techniques. *Chest*, 99:742–3.

Pastorino, U., Infante, M., Maioli, M., Chiesa, G. *et al.* (1993) Adjuvant treatment of stage I lung cancer with high dose vitamin A. *J. Clin. Oncol.*, 11:1216–1222.

Policard, A. (1924) Etude sur les aspects offerts par des tumeurs experimentales examinees a la lumiere de Wood. Comptre-rendus *Soc. Biol.*, 91:1423–24.

Ronchese, F. (1954) The fluorescence of cancer under the Wood light. *Oral. Surg. Oral. Med. Oral. Pathol.*, 7:967–971.

Sutro, C. J. and Burman, M. S. (1933) Examination of pathogenic tissue by filtered ultraviolet radiation. *Arch. Path.*, 16:346–349.

Thomas, P., Rubinstein, L. and the Lung Cancer Study Group (1990) Cancer recurrence after resection (TINO non small-cell lung cancer). *Ann. Thorac. Surg.*, 48:242–247.

Woolner, L. B. (1983) Pathology of cancer detected cytologically. In: *Atlas of early lung cancer*, National Cancer Institute Cooperative Early Lung Cancer Group, Igaku-Shoin, Tokyo, New York., pp. 107–213.

Woolner, L. B., Fontana, R. S., Cortese, D. A. *et al.* (1984) Roentgenographically occult lung cancer: Pathologic findings and frequency of multicentricity during a 10-year period. *Mayo. Clin. Proc.*, 59:453–466.

